# Supplementary material for: Helpful assistant or fruitful facilitator? Investigating how personas affect language model behavior
Source: PLoS One. 2025 Jun 30;20(6):e0325664. doi: 10.1371/journal.pone.0325664 (PMC12208479; doi:10.1371/journal.pone.0325664)
Supplement: Attachment — Submitted filename: responses_only.pdf [file pone.0325664.s004.pdf]

Response to the reviewer's comments

Pedro Henrique Luz de Araujo Benjamin Roth

February 27, 2025

This document includes a verbatim reproduction of the comments of each of the reviewers and our responses to each of the points raised. We are grateful for the constructive suggestions and hope this revision adequately addresses all concerns.

Reviewer 1

```
Thanks for the opportunity to read and comment on this paper. I think this is an
↪  interesting and certainly relevant study that could be published after some
↪  revisions have been made.

Major comments
1) Selection of LLMs: The authors use seven different large language models in
↪  their analysis --- seven out of a much larger collection of potentially
↪  relevant LLMs contained in the Transformers library. Unfortunately, the
↪  manuscript does not provide a clear explanation of justification for the
↪  selection of just these models. Why not one of the Llama models, for
↪  example? Case selection --- in this case, LLMs are different cases to be
↪  studied --- can have massive effects on a study's results, so the authors
↪  choices here really need to be made transparent and justified. Readers
↪  usually want a very convincing assurance that the selection of just these
↪  models does not skew the results in any way.
```

Response: We agree that selecting LLMs is a critical decision. Our selection rationale was to be as diverse as possible, given the resource constraints. That is why we include models from different families (GPT, Gemma, Mistral, and Zephyr), sizes (from 2B to dozens of billions of parameters), architectures (dense and mixture-of-experts models), and both open and closed-weight models. We have added this discussion in Section 3.

```
2) Human coders: To investigate their RQ3, the authors compare simulated LLM
↪  attitudes and perceptions of racism and toxicity of a set of tweets to
↪  corresponding data from humans. The problem here is that the authors only
↪  vaguely refer to a previous study from where the human data were (seemingly)
↪  taken (?), which this is not enough. The origin and attributes of the human
↪  attitude & tweet evaluation data need to be explained in more detail so that
↪  readers can understand what the authors are working with, what the potential
↪  weaknesses or shortcomings of these data are, and how the choice for those
↪  data could potentially influence the authors' results (are there
↪  alternatives?). All this should be transparent even when one has not read
↪  the other study in question.
```

Response: We thank the reviewer for pointing that out. We have added to Section 6.1 more details about the provenance of the questionnaires and tweet data. We have also included the questionnaires as supplementary material.

```
Minor comments
1) The graphs appear very grainy on my end and are difficult to read. Please
↪  upload them in a higher resolution and/or different file format.
```

Response: This issue arises from how PLOS One's submission system processes the manuscript PDF. The original pictures have high resolution and can be downloaded by clicking the link at the top of each preview page.

```
2) Some of the real-life personas that are used in the analyses (e.g., Rosa
↪  Luxemburg, Jörg Haider) are not introduced. I would not assume that everyone
↪  knows who Rosa Luxemburg was, and Jörg Haider is likely an even bigger
↪  unknown to many outside of (central/German-speaking) Europe. I would
↪  introduce them at least briefly in footnotes.
```

Response: PLOS One does not allow footnotes, and explaining each of the 27 political personas in the main body of the paper would be too verbose and detract from the text's main points. That said, we acknowledge the reviewer's point and have added brief explanations for the less-known real-life personas that are explicitly mentioned in the manuscript's discussions (Sec. 7).

```
3) On page 2, I would briefly define the term "toxicity"---it does become clear
↪  what this means from the context, but I still briefly stumbled over the term
↪  when reading.
```

Response: We thank the reviewer for the feedback. We have updated the Introduction to clarify what we mean by "toxicity": content that is harmful, offensive, or reproduces societal biases and stereotypes.

Reviewer 2

```
• Line 6 "burst the research" is a non-scientific expression. Rephrase it.
```

Response: We thank the reviewer for the suggestion. We have reformulated the passage.

```
• Line 78 - 90. This conclusion appears soon after the rational of the study,
↪  which reflect a jump for the reader. It appears not at a right place. Also
↪  recheck the claim language, 'first study to comprehensively investigate' the
↪  impact of personas on LLM behaviour". There are few published studies,
↪  please check if scope of the study similar or different.
```

Response: We have revised the passage to strengthen the connection between the study rationale and its contributions. We have also clarified that the manuscript describes, to the best of our knowledge, the first study to comprehensively analyze personas's influence on LLM behavior in different axes of behavior.

```
• Line 94 to 99. Please add more references.
```

Response: Following the reviewer's suggestion, we have added additional references.

```
• Line 254 - 257 how did you infer that statement? Can you show statistics in
↪  parenthesis?
```

Response: That is shown in Table 3: Domain-expert personas are ranked higher than out-of-domain personas. For example, technology personas have a top rank of 11 in STEM questions, while Law, Psychology, and Healthcare personas have top ranks of 87, 122, and 65. We have modified the passage to clarify these results.

- `Can you please indicate where Persona Configuration or variable details are ↪ mentioned in this article?`

Response: We list all personas and corresponding categories in Table 2. Table 1 lists examples of prompts for each dataset.

- `Can you please indicate what was the criteria for Personas programming and ↪ testing?`

Response: We use a persona set developed in previous work [9]. We selected this one due to the high number of different personas (163) and the diversity of persona categories (12 distinct categories).

- `Did you use any Scale of measuring 'the user satisfaction' If yes can you ↪ indicate, if NO can you indicate why not?`

Response: There is no "user satisfaction" measure in our experimental setting. All of our evaluations and research questions are formulated with respect to model responses on objective (TruthfulQA, BBQ, and MMLU datasets) or subjective (attitudes and toxicity datasets) datasets.

Reviewer 3

```
Dear authors,

Your article titled \Helpful assistant or fruitful facilitator? Investigating
↪ how personas affect language model behavior" addresses an important and
↪ timely topic, exploring the impact of assigned personas on the behavior of
↪ large language models (LLMs). The study is well-structured, clearly written,
↪ and provides valuable insights into the ways personas influence LLM outputs
↪ across various dimensions, including task performance, biases, social
↪ attitudes, and refusal rates.

Summary of the paper
Your paper investigates how assigning different personas to LLMs affects their
↪ responses across multiple dimensions. Specifically, you assign 162 personas
↪ from 12 categories (e.g., gender, sexual orientation, occupation) to seven
↪ different LLMs and prompt them with questions from five datasets. These
↪ datasets cover both objective (e.g., history, math) and subjective tasks
↪ (e.g., beliefs, values). The paper introduces a control persona setting
↪ (paraphrases of "helpful assistant") and an empty persona setting to isolate
↪ the effects of assigned personas.
The findings demonstrate that persona assignments lead to significant
↪ variability in responses across models, with some patterns of persona
↪ behavior generalizing across LLMs. You provide a rigorous quantitative
↪ analysis of task performance, social biases, and model refusal rates. The
↪ work contributes meaningfully to the literature by highlighting the
↪ inconsistencies in persona effects, particularly in how LLMs respond
↪ differently to personas with similar demographic traits.

Suggested revisions
```

```
1. Clarification on persona effects: While the results effectively show that
↪  different personas yield varying performances across models, an expanded
↪  discussion on why these differences occur would strengthen the argument. How
↪  could architectural differences between LLMs (e.g., ChatGPT vs. Mixtral)
↪  contribute to these disparities? What implications might these differences
↪  have for future model updates? This discussion would help clarify whether
↪  the differences stem from training data, fine-tuning methodologies, or other
↪  factors.
```

Response: The reviewer raises an interesting point. Our experiments include diverse LLMs (from different model families, sizes, and architectures) because we wanted to identify *what* are the effects of persona usage and *which* effects generalize across models. We have updated the conclusion to include a discussion on the open question of *why* personas yield varying performances.

```
2. Contextualization of refusals: The paper provides compelling evidence of
↪  disparities in refusal rates across personas. However, a deeper exploration
↪  of the ethical and practical implications of these refusals would be useful.
↪  How do these refusal patterns impact fairness, safety, and trust in LLMs?
↪  Discussing these concerns would provide a stronger contextual grounding for
↪  your findings.
```

Response: We thank the reviewer for this suggestion. We have expanded Section 7 to highlight and exemplify the implications of disparate refusals on model fairness, safety, and trustworthiness.

```
3. Recommendations for stakeholders: Given the study's findings, it would be
↪  beneficial to provide explicit (short) recommendations for different
↪  stakeholders:
o Model developers: How should developers address inconsistencies in persona
↪  performance and biases?
o Policy and safety regulators: What considerations should be made in regulating
↪  persona-based interactions in LLMs?
o Users: How can users be made aware of potential biases in persona-based LLM
↪  interactions?
```

Response: We thank the reviewer for the thoughtful feedback. We have updated the conclusion to include concise recommendations for model developers, regulators, and users.

```
4. Minor suggestions for clarity and rigor:
a) Clarification on Sample Size: It is unclear to me how many times each
↪  question was submitted per persona-model combination. Given that LLMs
↪  generate probabilistic outputs, could running multiple iterations (e.g., 50
↪  per persona-model pairing) and using modal responses improve robustness? If
↪  this was done, please clarify in the text; if not, discuss whether it could
↪  be a useful methodological refinement for future research.
```

Response: Each question was generally submitted once for each persona-model combination, except the attitude questionnaires, for which we generated 31 responses per data point (see Sec. 6.1). Contrary to the other four datasets, which contain hundreds or thousands of samples, the original attitude questionnaires only contained 27 items, which could be too few to measure the attitudes robustly. That is why we generated 30 additional responses for each item using paraphrased instructions (Table 8) and averaged the returned values as the final response for each original item. This resulted in a total of 837 responses per persona-model combination for the attitude questionnaires. We have clarified the sample size in Section 3.

Given the scale of our experiments, computing 50 responses per persona-model instance would be prohibitively expensive, as the current setup includes **90 million LLM generations** and

required significant GPU resources. However, we acknowledge that conducting a prompt sensitivity analysis could yield valuable insights for future research, and we have included this point in the conclusion.

```
b) Impact of system messages: Section 3 mentions some models receive system
↪  messages while others do not. Could this distinction introduce biases? Would
↪  the same models produce different results if all were tested with or without
↪  system messages?
```

Response: The only models that do not receive system messages are those that do not support that type of messaging. We always use system messages when possible as previous results suggest that models are more easily steered through system messages [24].

```
c) Control persona paraphrases: Table 2's control personas include some
↪  paraphrases (e.g., \competent second-in-command") that may not be
↪  semantically equivalent to \helpful assistant". Could this introduce
↪  unintended variation in results? Discuss whether this might contribute to
↪  arbitrary or disparate findings that are discussed later in the paper.
```

Response: The control personas do vary in their similarity to *helpful assistant*, which is expected given the large number of control personas (30). However, this variation does not undermine our findings. Our key result for control personas is that they exhibit lower variability in refusals than regular personas, even though some variation exists within the control set itself.

Regarding using control persona refusal variance as a baseline (Sec. 7.1), a stricter, less diverse control set could lead to more persona categories being flagged as having disparate refusals, potentially weakening the criterion. In contrast, using a more diverse control set strengthens confidence that the observed disparities arise from the personas themselves rather than from other factors, such as prompt sensitivity or random fluctuations.

```
d) Interpretation of Table 3: The sentence on lines 254-255 states "Table 3
↪  shows..." but does not explain why the results appear as they do. Also,
↪  Table 3 suggests that, overall, technology personas outperform others. Are
↪  these differences statistically significant? Providing an explanation might
↪  strengthen the interpretation.
```

Response: We have modified the text to contextualize Table 3 results better. We have also added to the text a report of a Wilcoxon signed-rank test showing that the difference between accuracies of in-domain and out-domain personas is statistically significant ($p < .05$).

```
e) Clarification in Tables 5, 6, 7: In row 490 and other references, should
↪  "homosexual" be replaced with "heterosexual" to align with the categories
↪  listed? "Gay" is already a category, but "heterosexual" does not seem to be
↪  listed.
```

Response: For tables 5, 6, and 7, we only include the personas with demographics represented in the BBQ dataset, as instances corresponding to the demographic are needed to compute self-bias and self-accuracy. There are no instances in BBQ corresponding to societal stereotypes against straight people, so it is not possible to include *straight person* in that analysis.

```
f) Statistical significance in Figures 6 and 9: Pearson correlation values are
↪  presented, but it is unclear which are statistically significant. If
↪  feasible and you feel this would add value to the results, consider
↪  highlighting coefficients that meet the 1% significance level with asterisks
↪  to improve interpretability (but this is a minor/optional suggestion).
```

Response: We are thankful for the suggestion. We have updated the figures to highlight significant coefficients ($p < .05$).
