# Supplementary material for: Helpful assistant or fruitful facilitator? Investigating how personas affect language model behavior
Source: PLoS One. 2025 Jun 30;20(6):e0325664. doi: 10.1371/journal.pone.0325664 (PMC12208479; doi:10.1371/journal.pone.0325664)
Supplement: Attachment — Submitted filename: Reviewer Comments.docx [file pone.0325664.s003.docx]

Manuscript Number: PONE-D-24-57315

Helpful assistant or fruitful facilitator? Investigating how personas affect language

model behavior

Order of Authors: Pedro Henrique Luz de Araujo

Benjamin Roth

**Reviewer Comments**

- Line 6 “burst the research” is a non-scientific expression. Rephrase it.
- Line 78 – 90. This conclusion appears soon after the rational of the study, which reflect a jump for the reader. It appears not at a right place. Also recheck the claim language, ‘first study to comprehensively investigate’ the impact of personas on LLM behaviour”. There are few published studies, please check if scope of the study similar or different.
- Line 94 to 99. Please add more references.
- Line 254 – 257 how did you infer that statement? Can you show statistics in parenthesis?
- Can you please indicate where Persona Configuration or variable details are mentioned in this article?
- Can you please indicate what was the criteria for Personas programming and testing?
- Did you use any Scale of measuring ‘the user satisfaction’ If yes can you indicate, if NO can you indicate why not?
